# Sustained MK-801 induced deficit in a novel probabilistic reversal learning task

**DOI:** 10.3389/fphar.2022.898548

**Published:** 2022-10-14

**Authors:** Patrick Latuske, Moritz von Heimendahl, Serena Deiana, Carsten T. Wotjak, Johann du Hoffmann

**Affiliations:** Central Nervous System Diseases Research, Boehringer Ingelheim Pharma GmbH & Co. KG, Biberach an der Riß, Germany

**Keywords:** reversal learning, cognitive flexibility, schizophrenia, drug discovery, dizocilpine (MK-801), behavioral task

## Abstract

Cognitive flexibility, the ability to adapt to unexpected changes, is critical for healthy environmental and social interactions, and thus to everyday functioning. In neuropsychiatric diseases, cognitive flexibility is often impaired and treatment options are lacking. Probabilistic reversal learning (PRL) is commonly used to measure cognitive flexibility in rodents and humans. In PRL tasks, subjects must sample choice options and, from probabilistic feedback, find the current best choice which then changes without warning. However, in rodents, pharmacological models of human cognitive impairment tend to disrupt only the first (or few) of several contingency reversals, making quantitative assessment of behavioral effects difficult. To address this limitation, we developed a novel rat PRL where reversals occur at relatively long intervals in time that demonstrates increased sensitivity to the non-competitive NMDA receptor antagonist MK-801. Here, we quantitively compare behavior in time-based PRL with a widely used task where reversals occur based on choice behavior. In time-based PRL, MK-801 induced sustained reversal learning deficits both in time and across reversal blocks but, at the same dose, only transient weak effects in performance-based PRL. Moreover, time-based PRL yielded better estimates of behavior and reinforcement learning model parameters, which opens meaningful pharmacological windows to efficiently test and develop novel drugs preclinically with the goal of improving cognitive impairment in human patients.

## Introduction

Cognitive deficits are observed in many neuropsychiatric disorders such as schizophrenia, attention deficit hyperactivity, obsessive-compulsive disorders, and autism spectrum disorders. In the clinic, the severity of cognitive impairment positively correlates with worse functional outcomes which suggests effective treatments will improve the quality of life of a patient (Green et al., 2000). Currently, pharmacotherapeutic options for neurocognition are limited which is a situation at least partially attributable to the difficulty of translating preclinical observations to human patients.

Cognitive flexibility, flexible adaptation to environmental change (Brown and Tait, 2015), is an important behavior that enables healthy social and environmental interactions. Probabilistic reversal learning (PRL) paradigms have been used to study cognitive flexibility in humans (Cools et al., 2002; Waltz and Gold, 2007), non-human primates (Costa et al., 2015; Rygula et al., 2015; Bartolo and Averbeck, 2020), and rodents (Ineichen et al., 2012; Dalton et al., 2014). Across PRL tasks and species, stimuli and choice feedback may differ, but fundamental task structures are similar. Subjects must sample several options and choices are rewarded with unique and unknown probabilities. To maximize reward (“win”), subjects must find the best option and then stick to it even after a non-rewarded choice. Once a learning criterion is reached, reward contingencies change. After contingency reversal, subjects must cognitively disengage from the previously better option and find the new best choice. This disengagement requires cognitive control processes which are often impaired in patients with psychiatric disorders (Waltz and Gold, 2007; Culbreth et al., 2016) or frontal lobe lesion (Hornak et al., 2004), and requires several brain regions including the orbitofrontal cortex, striatum, amygdala, and thalamus (O’Doherty et al., 2003; Hampton et al., 2007; Minzenberg et al., 2009; Klanker et al., 2013b).

To identify neuronal substrates supporting flexible choice in preclinical species, PRL tasks are often paired with a pharmacological challenge to model cognitive impairments observed in patients. For example, the NMDA receptor antagonist dizocilpine (MK-801) is known to induce cognitive deficits, impair reversal learning in rodents (van der Meulen et al., 2003; Dix et al., 2010; Svoboda et al., 2015; Savolainen et al., 2021), and induce c-Fos expression in brain areas implicated in the pathophysiology of schizophrenia (Dragunow and Faull, 1990; Väisänen et al., 2004). Reversible inactivation or lesion of brain areas has identified neural circuits that support cognitive flexibility in PRL tasks (Stalnaker et al., 2007; Rudebeck and Murray, 2008; Izquierdo et al., 2013; Dalton et al., 2016; Nakayama et al., 2018). However, in most serial reversal paradigms, these manipulations impair only the first (or few) contingency reversals (van der Meulen et al., 2003; Boulougouris et al., 2007; Klanker et al., 2013a; Dalton et al., 2016). Thus, many PRL tasks yield limited data, and the effects can be difficult to interpret from the perspective of neural circuit pharmacology and physiology. These limitations lead to small effects and large group sizes, and make the acquisition of meaningful physiological signals challenging. Critically, single reversal impairment leaves a small therapeutic window for pharmacological rescue of cognitive impairments by novel drugs designed to improve human cognition. Here, we addressed these limitations with a novel time-based PRL task and show that, unlike a widely used performance-based task (Floresco et al., 2008; Bari et al., 2010; Ineichen et al., 2012), MK-801 impairs task performance across several reversals.

## Methods

### Animals

Forty-four 280–300 g male Lister hooded rats from Charles River Germany were group-housed (2-4/Makrolon type-IV cage, standard enrichment) on a reverse 12 h light/dark cycle with controlled temperature (20°C–24°C) and relative humidity (∼45%–65%). Upon arrival, animals were fed *ad libitum* for 1 week and then habituated to handling and food restricted to ∼90% of their free-feeding weight. Experiments were conducted in the dark phase and in accordance with German animal welfare legislation, Association for Assessment and Accreditation of Laboratory Animal Care (AAALAC) regulations, and the USDA Animal Welfare Act, and approved by the Local Animal Care and Use Committee (18-017-G).

### Behavioral training

All behavior took place in operant boxes (Med Associates) controlled by custom MEDState scripts. Boxes were equipped with an illuminable food receptacle flanked by two retractable levers, cue lights above each lever, and a house light high above the receptacle. A photobeam measured receptacle entry and exit times. A pellet dispenser outside the chamber was used to deliver 45 mg sugar pellets (5TUL, TestDiet) into the receptacle. Behavioral events were recorded with a resolution of 1 ms.

### Performance-based probabilistic reversal learning

First, 14 animals were trained to enter the food receptacle after concurrent illumination and pellet delivery, which triggered a 10-s delay before the next pellet was dropped, and this was repeated 200 times. In stage two, both levers extended and a press at either lever was rewarded. Subsequent reward receptacle entries triggered a 5-s inter-trial interval (ITI), and the session ended with a maximum of 200 rewards. Once lever pressing stabilized, stage three required a nose poke at the illuminated food receptacle which triggered extension of both levers. After the lever was pressed, levers retracted and the reward was delivered. In stage four, a single lever was extended for 50 trials, after which the active lever was switched, and this pattern continued for 200 trials, which helps prevent side bias. To accustom animals to probabilistic reward, 80% of lever presses were rewarded. On unrewarded trials, the house light came on for 10 s before the next trial. In stage five, optimal and suboptimal levers were randomly and programmatically determined at the beginning of each session. Both levers were extended, and responses were rewarded with probabilities of 80% (optimal) and 20% (suboptimal), respectively. After reward collection, a 5-s ITI was triggered, but on non-rewarded trials, the house light switched on for 10 s. Reward contingencies were reversed (i.e., the optimal lever became suboptimal and *vice versa*) after eight consecutive responses on the optimal lever (Bari et al., 2010). To reduce predictability, 0–4 trials were added after 8 consecutive correct responses but before reversal. Sessions ended after 60 min, and the learning criterion was reached when animals made >10 reversals in a session.

### Time-based probabilistic reversal learning

In a separate cohort, training of 30 animals was identical to that of performance-based PRL, except there was one additional training stage. In the time-based schedule, reward contingencies were reversed randomly in time (∼10 min per block). Reversal times were drawn from a list without replacement (540, 480, 600, 660, and 720 s) for 6 total blocks and a session time of 60 min.

### Drugs

Experiments were within-subject where each animal received both the drug and vehicle on different days. On experimental days, 0.045 mg/kg (freebase) MK-801 [(+)-MK-801 hydrogen maleate, CAS 77086-22-7, Sigma-Aldrich] was dissolved in saline and half the cohort received this or a saline control subcutaneously 15 min before placing the animals in the chambers. On the following experiment day, treatment groups were reversed. Drugs were administered on Tuesdays and Fridays, followed by a washout day with no behavioral training.

### Trials to criterion

**FIGURE 1:**
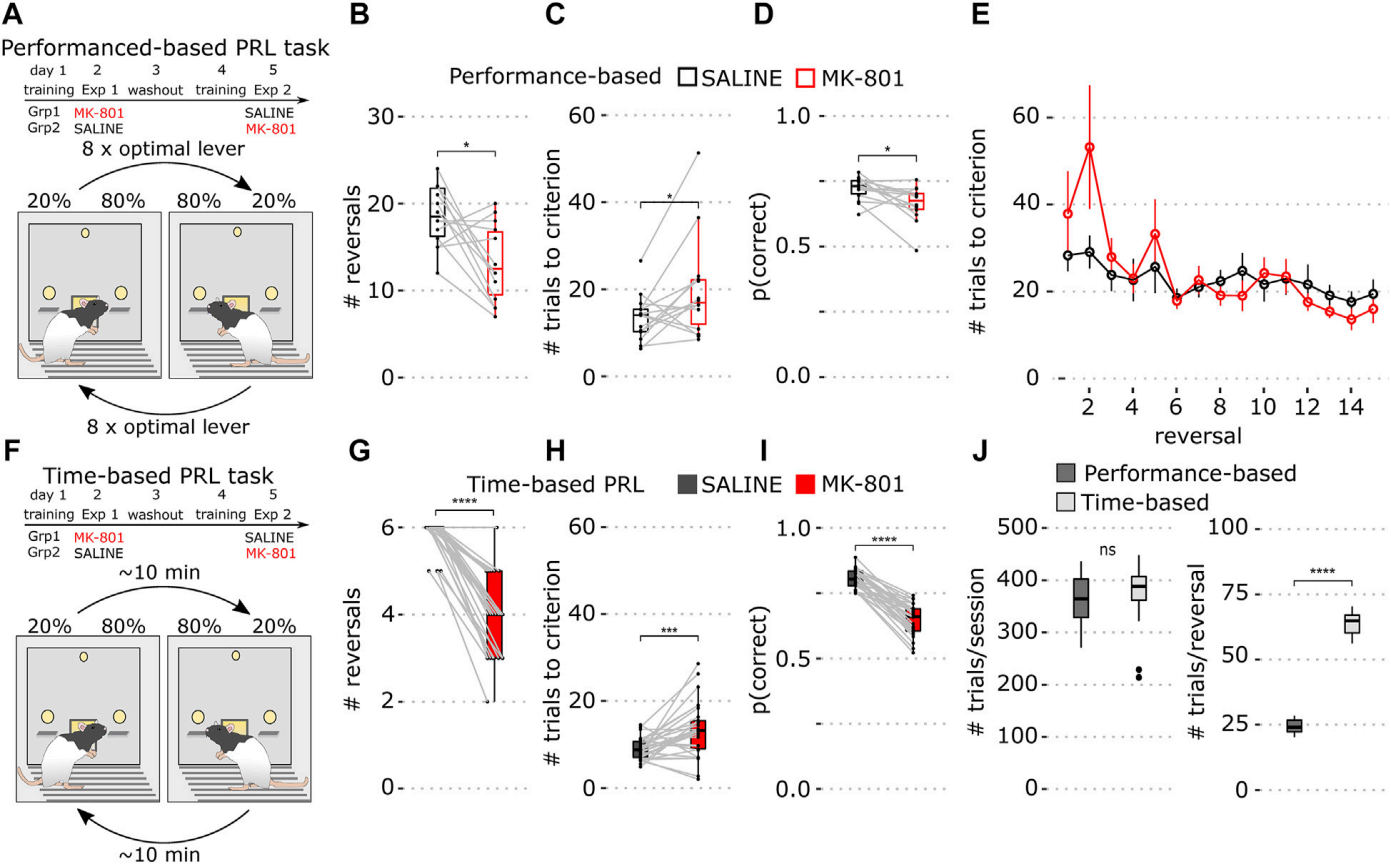
Performance- and time-based reversal tasks. **(A)** Schematics of the experiment and the performance-based PRL task schedule where reversals are triggered by 8 responses on the optimal lever. **(B)** Number of completed reversals in vehicle (black) and MK-801 (red) in the performance-based PRL. **(C)** Mean number of trials to criterion over all reversals in the performance-based PRL. **(D)** Proportion correct responses over the whole 60-min session **(E)** Number of trials to criterion for individual reversals. Data show mean ± SEM. **(F)** Schematic of the time-based PRL task schedule where reversals occur on an average every 10 min. **(G)** Number of completed reversals determined with a performance criterion (>8 consecutive correct responses) in the time-based PRL. **(H)** Mean number of trials to criterion across all reversals in the time-based PRL. **(I)** Average proportion of correct lever presses in the time-based PRL over the whole session. **(J)** Whisker plots of mean baseline parameters for performance-based (dark gray) and time-based PRL (light gray). Left: number of total trials; right: number of trials within reversals. For all figures, saline is shown in black and MK-801 in red. Whisker plots show the median and inner quartiles and whiskers extend the 1.5x interquartile range. *p < 0.05, ***p < 0.001, ****p < 0.0001.

To calculate trials to criterion (Figures 1C,E,H), trial number to 8 consecutive optimal lever presses was determined for each animal on each reversal, 8 was subtracted from this count, and these values were averaged across reversals. For Figure 1E, all animals achieved >6 reversals, but if later reversals were not completed they were excluded from those reversal means.

### Proportion correct response smoothed averages

**FIGURE 2:**
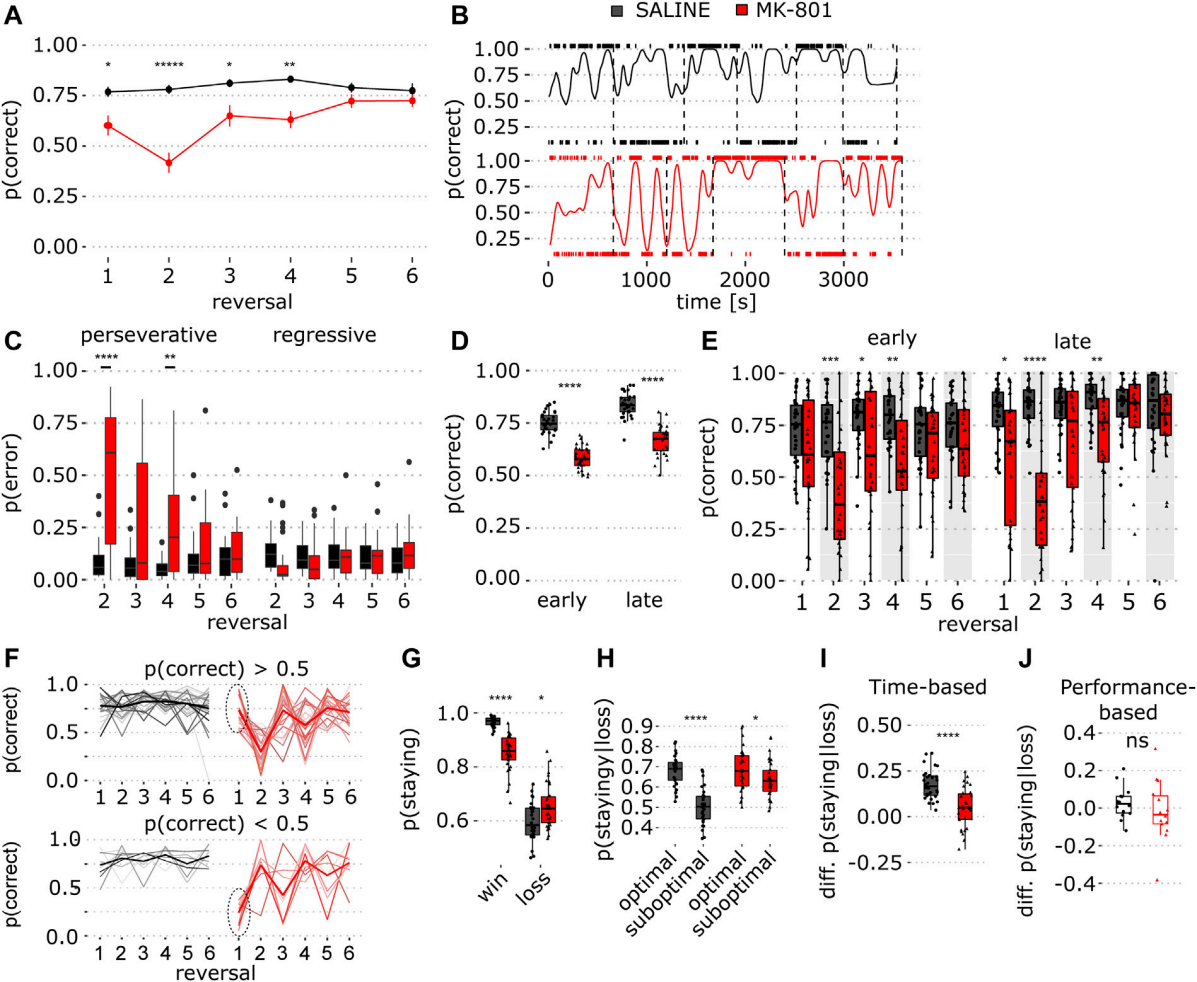
Sustained reversal deficit in time-based PRL in MK-801. **(A)** Proportion of correct responses in saline (black) and MK-801 (red) for individual reversal blocks. Data show mean ± SEM. Note the sustained effect of MK-801 in multiple reversals (red line, blocks 2-4) (for stats, see Table 1). **(B)** Single rat’s performance; trace shows a smoothed proportion of correct lever responses in saline (top) and MK-801 (below). Tick marks above and below each graph indicate right and left lever presses, respectively. Vertical dashed lines indicate reversals. **(C)** Proportion of perseverative and regressive errors in reversal blocks 2–6 (for stats, see Table 1). **(D)** Early and late trial performance measured as the proportion of correct lever presses collapsed over the whole session. **(E)** Same as in D, but for individual reversal blocks (for stats, see Table 1). **(F)** Proportion of correct responses for individual animals, stratified based on MK-801 block 1 performance (*p* (correct) > 0.5, top and *p* (correct) < 0.5 bottom). Dashed circles indicate selection criterion. **(G)** Probability of staying at the same lever after rewarded (win) and unrewarded trials (loss). **(H)** Conditional probability to stay after loss trials dependent on optimal and suboptimal choice. **(I)** Choice preference for optimal and suboptimal levers measured as the difference between the conditional staying probability (optimal–suboptimal) shown in **(H)**. **(J)** Same as J, but for performance-based PRL. *p < 0.05, **p < 0.001, ****p < 0.0001 (see Table 1 for statistical results).

To obtain continuous performance estimates for the proportion of correct responses (Figure 2B), binary response vectors were smoothed with a Gaussian kernel (ksmooth function, Base R, bandwidth = 9) with kernel estimates computed for every response time which reflects a weighted smoothed average of response vectors over time.

### Early and late trial performance

To estimate performance in early and late trials within blocks (Figures 2D,E), we counted within-block trials, divided these in half, and calculated the proportion of correct responses in the first and second halves of each block. Next, we averaged these within-phase values for each animal across all blocks (Figure 2D).

### Error analysis

In Figure 2C, within-animal regressive and perseverative errors were determined for blocks 2–6 based on choice history. Suboptimal lever presses were considered regressive errors after >8 consecutive responses on the optimal lever, while earlier suboptimal lever responses were considered perseverative errors.

### Probability of staying

In Figure 2G, win-stay and lose-stay probabilities were calculated for each animal by flagging responses on the same lever after rewarded (win) or unrewarded (loss) trials and dividing this number by the total number of win or loss trials, respectively. For Figure 2H, correct lose-stay and incorrect lose-stay probabilities for each animal were calculated for optimal or suboptimal levers.

### Reinforcement learning models

Four models were fit to data from both PRL tasks. Model 1 was a Rescorla–Wagner Q-learning model where the reward value of a choice *Q* ^*t*^_k_ is updated on trial *t*:

$$Q_{t+1}^{k}=Q_{t}^{k}+\text{α}\left(r_{t}-Q_{t}^{k}\right)$$

where $Q_{t}^{k}$ is the reward expectancy of choice k at trial t, *α* is the learning rate, and r is the reward. The probability of choosing option *k* is expressed in the softmax function:

$$p_{t}^{k}=\frac{\exp\left(\text{β}Q_{t}^{k}\right)}{\sum_{i}^{k}\exp\left(\text{β}Q_{t}^{i}\right)}$$

Model 2 included a stickiness parameter for repeating previous choices (Verharen et al., 2020). For Model 2, softmax was

$$p_{t}^{k}=\frac{\exp\left(\text{β}Q_{t}^{k}+{\text{θ}}^{k}\text{φ}\right)}{\sum_{i}^{k}\exp\left(\text{β}Q_{t}^{i}+{\text{θ}}^{i}\text{φ}\right)}$$

where *θ* is 1 if the current choice is the same as the previous trial and 0 otherwise, and *φ* is the weight to repeat the last choice.

Model 3 included a bias term for the left or right lever, which accounts for side bias. The softmax function was identical to that of Model 1, but Q-bias was added in each trial *t* to the value of $Q_{t}^{1}$ so that positive Q-bias values favor choice 1 and negative values favor choice 2 (Wilson and Collins, 2019).

Model 4 was a win-stay/lose-shift model with noise (∈) (Wilson and Collins, 2019). Responses depended only on previous feedback. Noise term ∈ added variability in the choice selection with the probability of choosing lever k.

if (lt−1=k and rt−1=1) OR (lt−1≠k and rt−1=0) → ptk=1−∈2

if (lt−1 ≠k and rt−1=1 ) OR (lt−1=k and rt−1=0)→ ptk=∈2

$l_{t-1}=1\mathrm{or}2$ for left or right levers at trial t-1, and $r_{t-1}\;=\;1\;\text{or}\;0$ for rewarded and unrewarded trials, respectively.

Models were fit in R (optimal function; BFGS method; fixed upper/lower bounds; L-BFGS-B), and bounds, based on the literature, were: *a* (0.1; 1.0), *β* (0.1; 10.0), *φ* (−5.0; 5.0), Q-bias (−1.0; 1.0), and ∈ (0.01; 1). Starting parameters were randomly selected and parameters fit to the training data of each animal using maximum likelihood. To compare models, we used the Bayesian information criterion (BIC) and the lowest score was selected as the winning model.

### Distribution of log latency values

**FIGURE 3:**
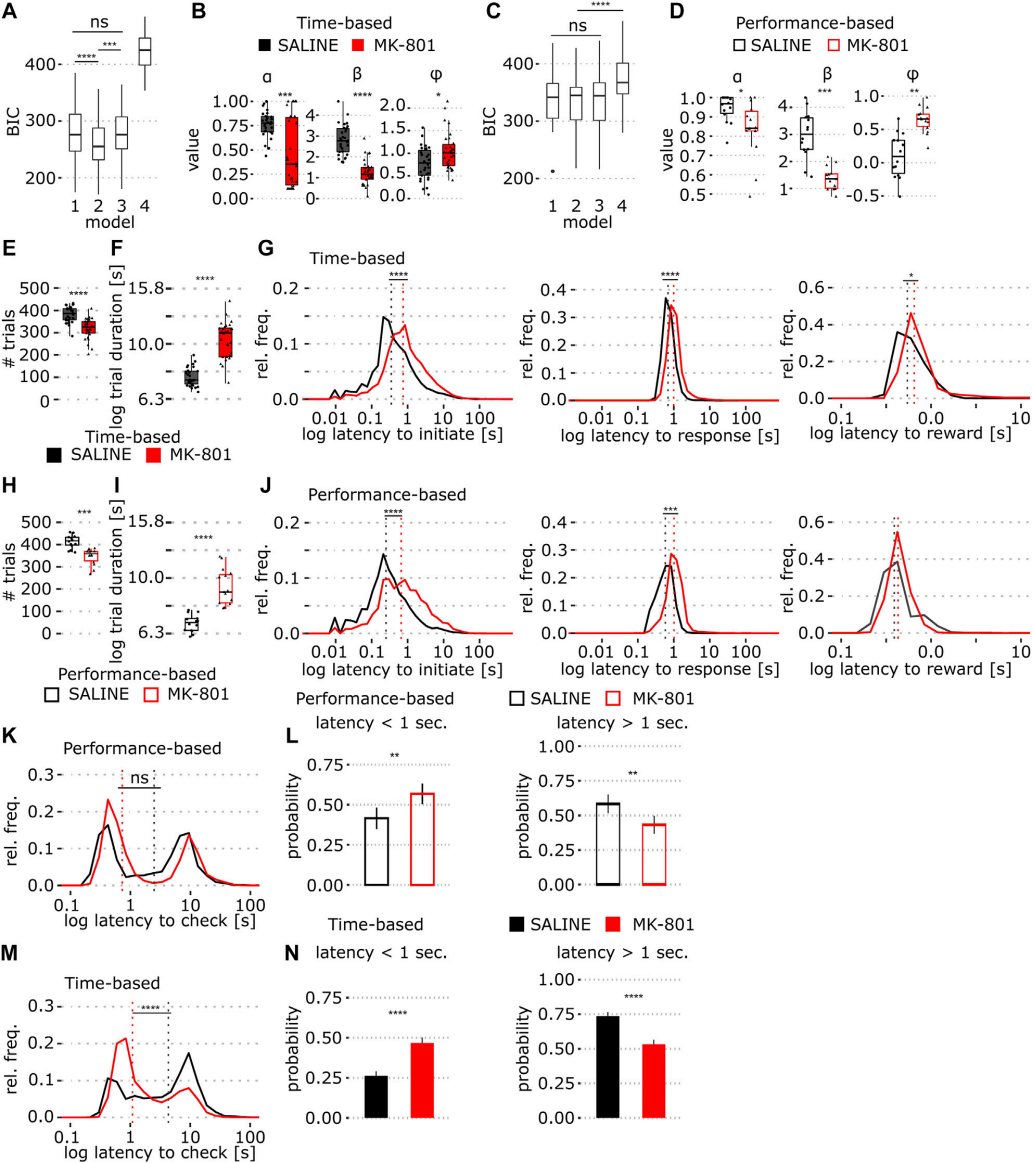
Comparison of behavior in time- and performance-based PRL tasks in MK-801. **(A)** BIC scores of four Rescorla–Wagner (RW) reinforcement learning models fit to the time-based PRL data; 1: standard RW, 2: RW + stickiness, 3: RW + side bias, and 4: win-stay/lose-shift model. **(B)** Model 2 coefficients (*α*, *β*, and *φ*) fit to the time-based PRL data. **(C,D)** Same as A and B, models were fit to performance-based task data. **(E)** Number of trials in the time-based PRL. **(F)** Time-based PRL mean trial duration across all trials (log10 transformed). **(G)** Time-based PRL log latency distributions of trial initiation (G, left), lever presses (G, middle), and reward collection (G, right). Dashed line indicates median values. **(H)** Number of trials in the performance-based PRL. **(I)** Performance-based PRL trial duration (log10 transformed). **(J)** Same as in G but for the performance-based PRL. **(K)** Time to magazine entry after unrewarded trials for the performance based PRL. **(L)** Proportion of short (<1 s, L, left) and long latency (>1 s, L, right) magazine entries on non-rewarded trials in the performance-based PRL (mean ± SEM). **(M,N)** Same as in K and L but for the time-based PRL task. Solid and empty boxplots show time- and performance-based task data, respectively. *p < 0.05, **p < 0.001, ****p < 0.0001 (see Table 1 for statistical results).

Latencies for trial initiation, lever press, reward collection (Figures 3G,J), and magazine entry after loss trials (Figures 3K,M) were log-transformed (log10). Trial initiation latency was the time from trial onset to the first subsequent receptacle entry. Lever press latency was the time after trial initiation to lever press. Reward collection and magazine entry after loss trials were the time from lever press to the first subsequent receptacle entry. Histograms (bin size 0.15 s) show within-condition pooled relative frequency distributions of log latency values from all animals on all trials. Vertical dashed lines indicate the median.

### Data analysis and statistics

Behavioral time stamps were analyzed and plotted with custom routines in R (version 3.6.1, R Core Team, 2019). Unless otherwise stated, paired Wilcoxon signed-rank tests were used for statistical comparisons. For mixed repeated measures ANOVA, within-subject factors were animal and reversal number (Figures 2A,C,E). To evaluate potential order effects, in a separate analysis for Figure 2A, the treatment day was included as an independent factor [treatment*order:F (1,28) = 4.98, *p* = 0.034], but *post hoc* comparisons were not significant (for details, see Table 1). The significance threshold for all statistical tests was set at *p < 0.05*.

**TABLE 1:** Summary statistics.

Figure		Variable	n	Measure 1		Measure 2		Test/Statistic	*p*-value	Adjusted	Signif.
										*p*-value (Holm)	
Figure 2	A	p(correct)		Saline	SE	MK-801		Paired Wilcoxon			
				Mean		Mean		signed rank test			
		1	30	0.77	0.03	0.6	0.05	368	0.004	0.016	*
		2	30	0.78	0.02	0.42	0.05	445	6.91e-7	4.15e-6	****
		3	30	0.81	0.02	0.65	0.05	366	0.005	0.016	*
		4	30	0.83	0.02	0.63	0.04	397	3.8e-4	1.9e-4	**
		5	30	0.79	0.02	0.72	0.03	299	0.08	0.16	ns
		6	30	0.77	0.04	0.73	0.03	316	0.088	0.16	ns
Figure 2	A	Order effects		Saline		Saline		Wilcoxon rank			
		of treatment		Day 1		Day 2		sum test			
			15	0.81	0.01	0.79	0.01	155	0.082		ns
				MK-801		MK-801		Wilcoxon rank			
				Day1		Day2		sum test			
			15	0.63	0.01	0.67	0.01	68	0.068		ns
Figure 2	C	p	n	Saline	IQR	MK-801	IQR	Paired Wilcoxon	*p*-value	Adjusted	Signif
		(perseverative errors)		Median		Median		signed rank test		*p*-value (Holm)	
		2	30	0.06	0.09	0.61	0.61	34	4.7e-5	2.32e-4	****
		3	30	0.06	0.09	0.08	0.56	112	0.023	0.069	ns
		4	30	0.04	0.06	0.20	0.37	73	6.1e-4	0.002432	**
		5	30	0.07	0.09	0.08	0.24	182.5	0.309	0.5	ns
		6	30	0.10	0.13	0.10	0.19	152	0.25	0.5	ns
		p	n	Saline	IQR	MK-801	IQR	Paired Wilcoxon	*p*-value	Adjusted	Signif
		(regressive errors)		Median		Median		signed rank test		*p*-value (Holm)	
		2	30	0.12	0.11	0.03	0.06	290	0.048	0.24	ns
		3	30	0.09	0.11	0.06	0.11	239	0.234	0.702	ns
		4	30	0.09	0.11	0.11	0.11	261	0.57	1	ns
		5	30	0.08	0.11	0.11	0.11	231	0.984	1	ns
		6	30	0.08	0.12	0.12	0.12	144	0.115	0.46	ns
Figure 2	E	p(correct)	n	Saline	IQR	MK-801	IQR	Paired Wilcoxon	*p*-value	Adjusted	Signif
		early		Median		Median		signed rank test		*p*-value (Holm)	
		1	30	0.76	0.24	0.61	0.42	318	0.08	0.154	ns
		2	30	0.77	0.25	0.37	0.42	424	1.8e-5	9.1e-5	****
		3	30	0.81	0.15	0.6	0.48	352	0.013	0.039	*
		4	30	0.8	0.21	0.53	0.34	384	0.001	0.004	**
		5	30	0.76	0.23	0.71	0.32	319	0.077	0.154	ns
		6	29	0.76	0.21	0.64	0.32				
		p(correct)	n	Saline	IQR	MK-801	IQR	Paired Wilcoxon	*p*-value	Adjusted	Signif
		late		Median		Median		signed rank test		*p*-value (Holm)	
		1	30	0.85	0.17	0.67	0.56	374	0.003	0.012	*
		2	30	0.87	0.14	0.38	0.35	453	1.3e-7	7.8e-7	****
		3	30	0.86	0.14	0.77	0.46	346.5	0.02	0.06	ns
		4	30	0.91	0.12	0.77	0.31	397	3.8e-4	0.0019	**
		5	30	0.87	0.13	0.86	0.21	252	0.7	0.7	ns
		6	30	0.87	0.26	0.81	0.2	276	0.21	0.42	ns
Figure 2	H	p(staying|loss)	n	Optimal	IQR	Suboptimal	IQR	Paired Wilcoxon	*p*-value		Signif
				Median		Median		signed rank test			
		Saline	30	0.69	0.09	0.5	0.11	465	1.86e-9		****
		MK-801	30	0.68	0.15	0.63	0.1	335	0.034		*
Figure 3	A	model	n	Median	model	Median	n	Paired Wilcoxon signed rank test	p-value	Adjusted	Signif
										p-value (Holm)	
		1	30	276.22	2	255.28	30	450	2.55e-7	1.54e-6	****
		2	30	255.28	3	276.23	30	50	5.59e-5	3.5e-4	***
		2	30	255.28	4	425.21	30	0	1.86e-9	1.12e-8	****
		1	30	276.22	3	276.23	30	217	0.761	1	ns
Figure 3	B	model		Saline	IQR	MK-801	IQR	Paired Wilcoxon	*p*-value	Adjusted	Signif
		coefficient		Median		Median		signed rank test		*p*-value (Holm)	
		α	30	0.78	0.16	0.36	0.695	403	2.0e-4	4.18e-4	***
		β	30	2.78	1.11	1.15	0.57	461	1.3e-8	3.9e-08	****
		φ	30	0.79	0.55	1.01	0.466	118	0.018	0.018	*
Figure 3	C	model	n	Median	model	Median	n	Paired Wilcoxon	*p*-value	Adjusted	Signif
								signed rank test		*p*-value (Holm)	
		1	30	341.85	2	345.24	30	63	0.542	1	ns
		2	30	345.24	3	344.44	30	37	0.358	1	ns
		2	30	345.24	4	367.6	30	3	6.1e-4	0.00366	**
		1	30	341.85	3	344.44	30	25	0.091	0.546	ns
Figure 3	D	model		Saline	IQR	MK-801	IQR	Paired Wilcoxon	*p*-value	Adjusted	Signif
		coefficient		Median		Median		signed rank test		*p*-value (Holm)	
		α	14	0.968	0.082	0.842	0.102	89	0.02	0.02	*
		β	14	3.01	1.165	1.357	0.532	102	6.1e-4	0.00183	***
		φ	14	0.093	0.496	0.656	0.185	5	0.001	0.002	**
Figure 3	G	Time-based	n	Saline	IQR	MK-801	IQR	Paired Wilcoxon	*p*-value	Adjusted	signif Signif
		PRL		Median		Median		signed rank test		*p*-value (Holm)	
		log latency to	30	−0.46	0.15	0.13	0.15	6	2.61e-8		****
		initiate									
		log latency to	30	−0.18	0.12	0.02	0.13	11	1.02e-7		****
		response									
		log latency to	30	−0.25	0.13	−0.21	0.11	288	0.017		*
		reward									
Figure 3	M	log latency to	30	0.59	0.46	0.08	0.25	459	2.61e-8		****
		check									
Figure 3	J	Performance	n	Saline	IQR	MK-801	IQR	Paired Wilcoxon	*p*-value	Adjusted	Signif
		based PRL		Median		Median		signed rank test		*p*-value (Holm)	
		log latency to	14	−0.62	0.15	−0.2	0.26	11	7.01e-5		***
		initiate									
		log latency to	14	−0.17	0.23	0	0.15	1	2.44e-4		***
		response									
		log latency to	14	−0.409	0.19	−3.67	0.14	27.5	0.124		ns
		reward									
Figure 3	K	log latency to	14	0.6	0.75	−0.13	1.14	67	0.391		ns
		check									

## Results

### Performance-based probabilistic reversal learning

First, we trained 14 rats on performance-based PRL with 80% and 20% reward probabilities where eight consecutive responses on the optimal lever triggered contingency reversals (Figure 1A). To assess pharmacological sensitivity, we compared performance in MK-801 and vehicle control.

In MK-801, rats showed a modest deficit primarily reflected in a reduced number of completed reversals (Figure 1B; *p* = 0.01), an increased number of errors to reach criterion (Figure 1C; *p* = 0.035), and fewer correct lever presses (Figure 1D; *p* = 0.01). However, due to high variability, trials to the criterion for individual reversals (Figure 1E, notably block 2) did not differ in MK-801 and control. Consistent with previous reports, MK-801 had little effect after the first reversal (van der Meulen et al., 2003; Kumar et al., 2015).

Due to these weak effects, we modified the task with the goal of increasing its pharmacological sensitivity. We reasoned that rapid switching of contingency may facilitate adaptive strategies like win-stay/lose-shift and thus reduce MK-801 sensitivity. To test this hypothesis, we implemented a time-based protocol where reward contingencies were reversed approximately every 10 min for 60 min, thereby increasing within-block trial numbers and reducing reversal frequency (Figure 1F; see Methods for details).

### Time-based probabilistic reversal learning

We trained 30 rats on the time-based PRL and found the total number of trials did not differ between tasks (Figure 1J; *p* = 0.093, Wilcoxon rank-sum test), but, as expected, the number of trials within block was increased in time-based PRL (Figure 1J; *p* = 1.7 × 10^−11^, Wilcoxon rank-sum test). *Post hoc* analysis of blocks 1–6 with the learning criterion (8 consecutive optimal lever presses) showed that animals learned slightly faster after reversal in time-based PRL (11.4 (time-based) vs. 13.8 (performance-based); *p* < 0.05, Wilcoxon rank-sum test, data not shown).

Next, in cross-over, we tested MK-801 sensitivity of time-based PRL. For direct comparison, we applied *post hoc* the performance criterion and found that MK-801 significantly reduced completed reversals (Figure 1G; *p* = 2.3 × 10^−13^) and increased trials to criterion (Figure 1H; *p* = 6.8 × 10^−4^). MK-801 also significantly reduced the proportion of correct responses compared to saline in the 60-min session (Figure 1I; *p* = 1.8 × 10^−9^) and these differences were more robust than those in performance-based PRL.

The analysis of individual reversal blocks showed that MK-801 reduced correct responses most profoundly in block 2, but this deficit was sustained for 3 reversals (Figure 2A; *p* = 1.82 × 10^−16^, two-way ANOVA, and Table 1). Next, we calculated a smoothed kernel average of the proportion of correct responses across the entire session. This showed that, after reversal, saline-treated rats responded predominantly to the previously optimal (but now suboptimal) lever (Figure 2B) and that this bias gradually shifted toward the newly optimal lever. However, this shift was not so pronounced in MK-801. To quantify this, we calculated within-block probability of perseverative and regressive errors (see Methods for details) and found that in MK-801, animals made more perseverative errors than in control (Figure 2C; *p* = 9.57 × 10^−13^, two-way ANOVA). The analysis of individual blocks revealed that MK-801 greatly increased perseverative errors in reversal blocks 2–4 (Figure 2C; left panel, see Table 1) with no effect on regressive errors.

To quantify within-block learning, we calculated the proportion of correct responses for early and late trials by halving reversal blocks based on the number of trials and compared performance in MK-801 and control (Figures 2D,E). In early trials, ANOVA revealed a main effect of treatment (Figure 2E; *p* = 1.27 × 10^−12^, two-way ANOVA). A *post hoc* comparison showed that MK-801 reduced the percentage of correct choices in reversal blocks 2–4 (see Table 1) and that initial discrimination (block 1) was unimpaired. In late trials, MK-801 reduced performance in reversal blocks 1, 2, and 4 (Figure 2E; *p* = 3.13 × 10^−13^, two-way ANOVA). In MK-801, we saw impaired average behavior in block 4 but not in late block 3 trials. To investigate this in detail, we stratified animals into two groups based on performance (*p* (correct) > 0.5 and *p* (correct) < 0.5) in MK-801 in the first block (Figure 2F). Interestingly, most animals (*n* = 22) with good performance in initial discrimination (block 1) exhibited profound impairment in blocks 2 and 4, whereas the smaller group (*n* = 8) was impaired in blocks 1 and 3. When averaged, the MK-801 induced side bias in alternating blocks drives reversal deficits in blocks 2–4. Taken together, unlike in performance-based PRL, where reversals can occur with a higher frequency, MK-801 impaired performance in time-based PRL up to reversal block 4 and, consistent with its pharmacokinetic profile, for ∼50 min (Wegener et al., 2011).

To understand how outcomes influence choice, we calculated conditional probabilities for choosing the same lever after rewarded and unrewarded choices. In MK-801, the probability to repeat choices after rewarded trials was lower than that in vehicle (Figure 2G; *p* = 1.9 × 10^−9^) and higher after unrewarded trials (Figure 2G; *p* = 0.011), suggesting that in MK-801 rats are less sensitive to reward-based feedback.

Previous reports suggest rats infer task features to optimize response strategies (Dhawan et al., 2019). Thus, we hypothesized that if rats tracked current reward probabilities, this should be reflected in staying probabilities for optimal and suboptimal levers after non-rewarded trials. Indeed, in saline, rats stayed more on optimal than on suboptimal levers after loss trials (Figure 2H; Table 1). Quantification of choosing the same lever after unrewarded trials showed that MK-801 reduced optimal lever preference in the absence of positive feedback (Figure 2I; *p* = 1.2 × 10^−5^). Interestingly, there was no difference in lever differentiation in performance-based PRL, suggesting different strategies may be employed in the two tasks (Figure 2J; *p* = 0.42).

Next, for direct comparison of strategy in the two tasks, we implemented a Rescorla–Wagner Q-learning model (Model 1) with extensions for stickiness (Model 2), or side-bias (Model 3) and a win-stay/lose-shift model (Model 4) that differentially integrates reward history relative to trial-by-trial choice. Using the Bayesian information criterion (BIC), we compared model fit to training data and found that Model 2 (with the stickiness parameter) best fit time-based PRL data (Figure 3A; *p* = 1.67 × 10^−20^, two-way ANOVA, see Table 1) and models 1–3 performed equally well for performance-based PRL (Figure 3C). Thus, we applied Model 2 to predict the learning rate (*α*), inverse temperature (*ß*), and stickiness (*φ*) in both tasks. We found a remarkable decrease in the learning rate in time-based PRL in MK-801 and weaker effects in performance-based PRL (Figures 3B,D). MK-801 also reduced *ß*, reflecting a decrease in value-driven choice (Figures 3B,D), and increased the tendency to repeat choices by increasing stickiness (Figures 3B,D; for stats Table 1). These results support the idea that MK-801 reduces reward sensitivity and increases perseverative behavior (Stefani and Moghaddam, 2005; Thonnard et al., 2019) but with larger effect sizes in the time-based protocol.

To examine whether reduced reward sensitivity reflects motivational deficits, we compared trial numbers between conditions and found that MK-801 reduced the total number of trials in time-based PRL (387 (saline) vs. 326 (MK-801), median; Figure 3E; *p* = 8.8 × 10^−6^). Consequently, we examined how MK-801 influenced processing and reaction times. In time-based PRL, MK-801-treated animals were slower to initiate trials, press levers, and collect rewards (Figure 3G; Table 1). These small, but significant, lengthening of latency accumulated to an average of 0.75 s per trial, which may partially account for reduced response rates in MK-801. Next, we calculated trial durations (time between initiation cues), which includes the 10 s after unrewarded trials that occur more in MK-801. In time-based PRL, MK-801 increased median trial length by 3.5 s compared to the vehicle (Figure 3F, median, 7.4 s (saline) vs. 10.9 s (MK-801); *p* = 9.75 × 10^−10^). In the performance-based PRL, we also observed a reduction in trial number (Figure 3H; *p* = 1.03 × 10^−4^), longer behavioral latencies (Figure 3J), and increased median trial lengths, albeit to a lesser extent (Figure 3I, 6.9 s (saline) vs. 8.9 s (MK-801); *p* = 4.99 × 10^−8^). Taken together, these results suggest MK-801 may not reduce motivation *per se* but rather induce motoric and/or attentional impairment leading to less lever pressing.

In saline and MK-801, receptacle latencies after non-rewarded choices were bimodal in performance-based PRL (Figures 3K,L), and both peaks were right shifted by MK-801. In contrast, in time-based PRL, MK-801 strongly shifted the distribution toward shorter latencies (<1 s; Figures 3M,N). To quantify this effect, which may reflect altered reward anticipation, we split the distributions into short (<1 s) and long (>1 s) latencies and calculated the relevant areas under the curve. In performance-based PRL, MK-801 induced shorter latencies than saline (Figures 3L; *p* = 0.004), but this shift was bigger in time-based PRL (Figures 3N; *p* = 1.3 × 10^−7^), where twice as many latencies were <1 s than in saline. These short-latency non-rewarded entries likely reflect false reward anticipation, while longer latency entries may be premature trial initiation attempts. These data suggest that in MK-801, animals falsely anticipate reward on loss trials, and this effect is bigger in time-based PRL.

## Discussion

Here, we developed a probabilistic reversal learning task where reversals occur pseudo-randomly in time at relatively long intervals and compared this paradigm to performance-based reversal learning in which reversals tend to occur with higher frequency. We found that MK-801 had weak transient effects on performance-based PRL (Figures 1D,E) but severely impaired time-based PRL across four reversal blocks (Figures 2A–F). These sustained deficits were accompanied by strong perseveration (Figure 2C) and impaired, but not broken choice-outcome association (Figures 2G,H). We found that time-based PRL had several advantages: first, greater time spent in each block and lower reversal frequency make it difficult for animals to adjust to sudden changes in reward contingencies; second, more within-block choices make estimation of learning and performance more reliable and robust. Furthermore, our results suggest rats use a different strategy in time-based PRL that renders behavior more sensitive to MK-801.

One potential explanation for increased MK-801 sensitivity is that time-based PRL requires sustained cortical and cognitive engagement. Indeed, in serial reversal tasks, pharmacological challenges and brain region-specific inactivation tend to impair the first of several reversals (Boulougouris et al., 2007; Young and Shapiro, 2009; Dalton et al., 2016). While the mechanism is unknown, animals may develop attentional-sets or rules rendering later reversals easier to solve which may be driven by reduction in choice sampling or overtraining (Mackintosh and Little, 1969; Dhawan et al., 2019). In time-based PRL, performance was impaired across four blocks (Figure 2A) indicating later reversals remained difficult in MK-801. These sustained deficits are likely due to longer inter-reversal times which prevent behavioral adaptations aimed at simplifying the task. Indeed, high-frequency reversals are less dependent on OFC (Riceberg and Shapiro, 2017) which suggests that low-frequency and high-frequency reversals engage the cortex differently. Consistent with this idea, we found that in time-based PRL optimal lever preference was maintained after unrewarded trials (Figure 2I) which was not the case in performance-based PRL (Figure 2J). Furthermore, our modeling results suggest that in time-based PRL rats did not follow a win-stay/lose-shift strategy (Figures 3A,C), but that choice value is integrated over longer periods of time. In contrast, in performance-based PRL where reward probabilities are less stable recent reward history is more relevant. This conclusion is further strengthened by lower learning rate (*α*) and higher stickiness coefficients (*φ*) in the time-based task suggesting lower value of recent reward (Figures 3A–D) (Zhang et al., 2020), and higher stickiness at the optimal lever irrespective of outcome (Figures 2H,I). In addition, a weak learning criterion of 8 optimal lever presses can be reached by chance and lead to 2–3 reversals in 400 trials (Metha et al., 2020). The possibility of some success with a random strategy likely increases data variability and reduces pharmacological effect sizes in performance-based PRL.

In Figure 2F, in MK-801, two-thirds of animals (*n* = 22) found the optimal lever in block 1 and then largely perseverated at that lever through block 4, while the remaining animals (*n* = 8) had an antiphase-like pattern of perseveration. This strong perseveration in blocks 2–4 (Figure 2C) makes it appear that performance improves in block 3 and then is impaired again in block 4. However, this is driven by reward contingency reversal where the optimal lever becomes the same as the one at which they chose to perseverate. Thus, apparently good performance in block 3 also reflects behavioral impairment. In most animals, discrimination in block 1 was good (Figure 2F), suggesting MK-801 effects in blocks 2–4 are reversal deficits driven by perseverative behavior consistent with sustained cognitive flexibility deficits.

Surprisingly, MK-801 reduced the number of trials in both tasks (Figures 3E,H). This was unexpected, as MK-801 induces hyperactivity, and in some tasks, it increases lever pressing (Gastambide et al., 2013). Here, in MK-801, animals were slower to initiate trials, press levers, and collect rewards (Figures 3G–J). In addition, MK-801 increased sub-optimal pressing (Figures 1D,I), and 80% of these choices lead to a 10 s timeout which reduces the time levers are available for pressing. Together, slower trial completion and more errors can account for the ∼14% reduction of responses in MK-801; this likely reflects generalized motoric and/or attentional delay and is not consistent with motivational deficits one might expect with specific impairments in reward approach and/or reward collection latency.

Previous studies have shown that cortex is differentially required depending on reward stability, reversal frequency (Riceberg and Shapiro, 2017) and the inclusion of irrelevant stimuli or non-rewarded choice options (Ragozzino, 2007). Related to this, our data suggest that time- and performance-based tasks likely engage neural circuits differently. Future studies will evaluate the cortical and subcortical neural circuits and neurotransmitter systems that support flexible choice behavior in the time-based PRL task. Our analysis suggests time-based PRL may facilitate drug discovery by allowing higher-throughput compound screening, acquisition of relevant physiological readouts for biomarker identification, and thereby aid discovery of novel and efficacious drugs to treat cognitive flexibility deficits in human patients.

## Data Availability

The raw data supporting the conclusion of this article will be made available by the authors, without undue reservation.
